# Histone acetylation associated up-regulation of the cell wall related genes is involved in salt stress induced maize root swelling

**DOI:** 10.1186/1471-2229-14-105

**Published:** 2014-04-23

**Authors:** Hui Li, Shihan Yan, Lin Zhao, Junjun Tan, Qi Zhang, Fei Gao, Pu Wang, Haoli Hou, Lijia Li

**Affiliations:** 1 State Key Laboratory of Hybrid Rice, College of Life Sciences, Wuhan University, Wuhan 430072, China

**Keywords:** *Zea mays*, Cell enlargement, Cell wall related genes, Histone acetylation, Root swelling, Salt stress

## Abstract

**Background:**

Salt stress usually causes crop growth inhibition and yield decrease. Epigenetic regulation is involved in plant responses to environmental stimuli. The epigenetic regulation of the cell wall related genes associated with the salt-induced cellular response is still little known. This study aimed to analyze cell morphological alterations in maize roots as a consequence of excess salinity in relation to the transcriptional and epigenetic regulation of the cell wall related protein genes.

**Results:**

In this study, maize seedling roots got shorter and displayed swelling after exposure to 200 mM NaCl for 48 h and 96 h. Cytological observation showed that the growth inhibition of maize roots was due to the reduction in meristematic zone cell division activity and elongation zone cell production. The enlargement of the stele tissue and cortex cells contributed to root swelling in the elongation zone. The cell wall is thought to be the major control point for cell enlargement. Cell wall related proteins include xyloglucan endotransglucosylase (XET), expansins (EXP), and the plasma membrane proton pump (MHA). RT-PCR results displayed an up-regulation of cell wall related *ZmEXPA1*, *ZmEXPA3*, *ZmEXPA5*, *ZmEXPB1*, *ZmEXPB2* and *ZmXET1* genes and the down-regulation of cell wall related *ZmEXPB4* and *ZmMHA* genes as the duration of exposure was increased. Histone acetylation is regulated by HATs, which are often correlated with gene activation. The expression of histone acetyltransferase genes *ZmHATB* and *ZmGCN5* was increased after 200 mM NaCl treatment, accompanied by an increase in the global acetylation levels of histones H3K9 and H4K5. ChIP experiment showed that the up-regulation of the *ZmEXPB2* and *ZmXET1* genes was associated with the elevated H3K9 acetylation levels on the promoter regions and coding regions of these two genes.

**Conclusions:**

These data suggested that the up-regulation of some cell wall related genes mediated cell enlargement to possibly mitigate the salinity-induced ionic toxicity, and different genes had specific function in response to salt stress. Histone modification as a mediator may contribute to rapid regulation of cell wall related gene expression, which reduces the damage of excess salinity to plants.

## Background

Soil salinization owing to agricultural irrigation leads to crop growth rate reduction and yield decrease. The understanding of the mechanisms by which plants cope with high concentration of salt could enhance productivity in the high saline conditions. Excess NaCl inhibits plant growth both in shoots and roots [1]. A significant growth reduction in the maize shoot and primary root is observed following NaCl treatment [2,3]. One reason of the growth suppression is inadequate photosynthesis due to stomatal closure and consequently limited carbon dioxide uptake under salt stress [4] and thus most morphological and transcriptional studies on the effect of excess salinity have been focused on shoots and leaves because they are responsible for photosynthesis. But the effect of this stress on roots should be more obvious as the root is the organ that is directly exposed to the salinity soil [5]. The molecular and cellular mechanism why the growth of young roots was repressed under salt stress is not precisely known.

The plant growth requires concerted water uptake and irreversible cell wall expansion to enlarge cells [6]. The mechanical character of the cell wall controls the cell size and shape through the governance of cell expansion, which determines the morphology of tissues and organs [7]. Several studies using transgenic materials have confirmed the role of expansins in promoting cell enlargement by affecting cell wall loosening [8,9]. The plant cell wall is a dynamic network structure that consists of cellulose microfibrils and helicellulose embedded in a pectin matrix and contains proteins and numerous enzymes [10]. This structure is important in plant growth and development and in response to various environmental stresses [11]. The cell wall related proteins are believed to play a role in modulating cell wall extensibility that mediates cell enlargement and expansion. These proteins include xyloglucan endotransglucosylase (XET), endo-1,4-b-D-endoglucanase (EGase), expansins (EXP), and the plasma membrane proton pump (PM-H^+^-ATPase, MHA) [12]. The low water potential is found to increase XET activity in the apical region of maize roots [13], although the possible role of XET in cell wall extension could not yet be confirmed in vitro [14]. Expansins have been reported to induce immediate cell wall loosening in vitro and in vivo [15], and may be involved in acid-induced growth through disrupting the link between cellulose microfibrils and adjacent matrix [16]. The expansin gene family that shares conserved motifs comprises four gene subfamilies: α-expansin (EXPA), β-expansin (EXPB), expansin-like A, and expansin-like B [17]. The expansin gene expression level is highly related with the elongation growth of roots, internodes and leaves [8,9,18]. However, individual expansins are observed to be prior expressed in specific organs, which suggested that individual expansin genes had specific roles for plant development. PM-H^+^-ATPase can pump protons into the apoplast from the cytosol to acidify the apoplast where acidification activates expansin activity that in turn loosens the cell wall and expands cells [19]. Xyloglucan is the most common hemicellulose in the primary cell wall in most plants. XET has been proposed as a potential cell wall extension protein because XET is able to cleave and rejoin xyloglucan chains [20]. An up-regulation of the *ZmXET1*, *ZmEXPA1*, and *ZmMHA* mRNAs is found in maize shoots [12].

The gene expression is influenced by chromatin structure, which is dependent on epigenetic regulation, such as histone post-translational modifications and DNA methylation. The basic repeated unit of chromatin is the nucleosome in eukaryotes, which is formed by wrapping approximately 146 bp of DNA around a histone octamer that consists of two copies of each histone proteins, H2A, H2B, H3 and H4 [21]. The N-terminus tail (N-tail) amino acid residue of the histones, exposed on the surface of the nucleosome, is subjected to post-translational modifications, including acetylation, methylation, phosphorylation and ubiquitination, catalyzed by histone modification enzymes [22,23]. Histone acetyltransferases (HATs) are classified into two categories based on their subcellular distribution: the type A HATs and the type B HATs [24]. Histone acetylation is regulated by HATs and often correlated with gene activation [25]. Histone modification is involved in transcriptional regulation of many genes under salt stress [26,27].

An understanding of the growth response of crop roots at cellular and molecular levels to salinity is of fundamental importance for a better comprehension of plant resistance to excess salinity and the breeding of salt stress-adapted crops. The cell wall is thought to be the major control point for cell enlargement, which is related with plant stress response. Currently, little is known about whether the histone modification is involved in regulating the expression of the cell wall related genes under salt stress conditions. This study aimed to analyze cell morphological alterations in maize roots as a consequence of excess salt in relation to the transcriptional and epigenetic regulation of the cell wall related protein genes. Salt stress induced maize growth inhibition along with root swelling and cell enlargement, which were accompanied by an up-regulation in some cell wall related genes. The global histone acetylation levels of H3K9 and H4K5 were increased in treated seedlings and the transcript levels of the *ZmHATB* and *ZmGCN5* genes were increased, which might be an adaptive response of plants to salt stress. ChIP results displayed that up-regulation of the *ZmEXPB2* and *ZmXET1* genes was associated with an increase in histone H3K9 acetylation levels on the promoter regions and coding regions of these two genes in response to salt stress. Our data indicated that salt stress-induced elevation of H3K9Ac was accompanied by the change of cell well related gene expression, resulting in an adaptive cellular and growth response.

## Results

### High salinity causes the elongation zone swelling and the meristematic zone shortening

**Figure 1:**
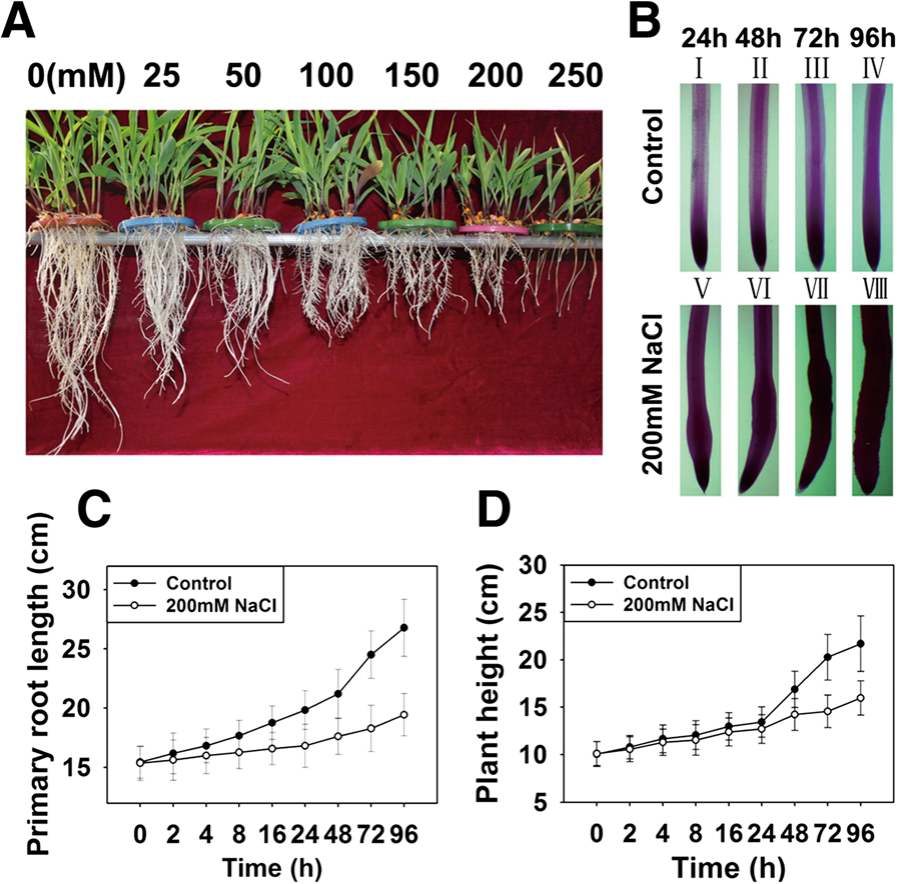
**Phenotype change of maize seedlings under NaCl stress conditions. (A)** Six-day-old maize seedlings were transferred to nutrient solution supplemented with 0, 25, 50, 100, 150, 200 and 250 mM NaCl respectively and grown for 7 days. The seedling and root growth was inhibited. **(B)** The primary roots from maize seedlings were stained with Schiff’s Solution. I-IV Six-day-old maize seedlings under normal growth conditions in nutrient solution for 24, 48, 72 and 96 h, respectively. V-VII Six-day-old maize seedlings treated with 200 mM NaCl in nutrient solution for 24, 48, 72 and 96 h, respectively. Each experiment was repeated three times. Roots were swollen at the elongation zone and the meristematic zone length was decreased after treatment with 200 mM NaCl as compared with the control group. Bar = 1 mm. **(C, D)** Six-day-old maize seedlings were transferred to nutrient solution supplemented with or without 200 mM NaCl, and the primary root length **(C)** and the plant height **(D)** were measured at the indicated time points. Compared with the control group, the primary root length and the plant height were dramatically reduced following exposure to 200 mM NaCl for 96 h. Values are mean values ± SD (the standard deviation) (n = 20).

**Figure 2:**
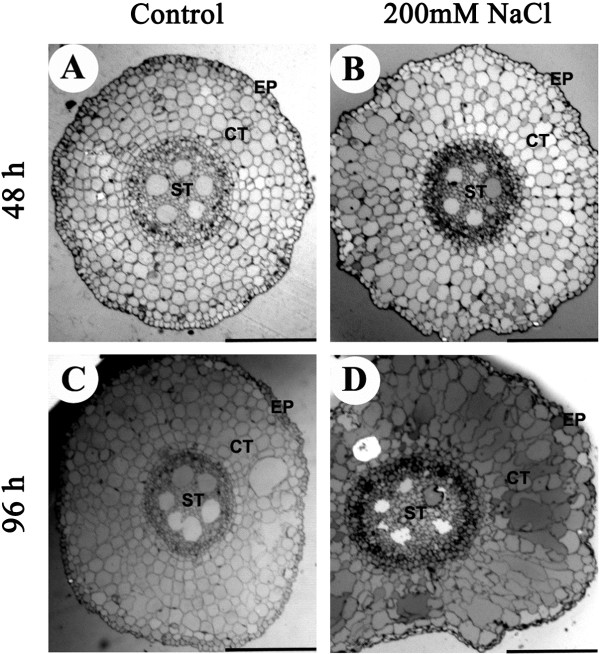
**Light-microscopy images of MB-stained transverse sections.** Transverse sections of six-day-old maize root tips grown under control conditions **(A, C)** or in the presence of 200 mM NaCl in nutrient solution **(B, D)** for 48 h **(A, B)** and 96 h **(C, D)**. The cortical cells were radially enlarged after stressed with 200 mM NaCl for 48 h and 96 h as compared with the control group. Photographs were taken under bright-field illumination. CT - cortex; EP - epidermal layer; ST - stele tissue. Bar = 0.25 mm.

Six-day-old maize seedlings were transferred to 1/2 Hoagland’s nutrient solution supplemented with different concentrations of NaCl and were further grown for 7 days, and the results showed that seedling growth was inhibited as well as the secondary root was reduced obviously in varying degrees (Figure 1A). As expected, 250 mM NaCl often cause leaves to wither and even die, and thus 200 mM NaCl was chosen for this study, also based on the reported result [28]. After exposure to 200 mM NaCl, the primary root got shorter, while roots were swollen at the elongation zone and the length of the meristematic zone was decreased. The swelling zone became longer with the increasing of the treatment time as compared with the control group (Figure 1B). Following 200 mM NaCl treatment, the primary root length and the plant height were dramatically reduced and after 96 h the primary root length was decreased by 27% (Figure 1C) and the plant height was reduced by 26% (Figure 1D) as compared with the control group. Next, we wanted to know cellular changes at the swollen region. The transverse sections of the swollen part in the elongation zone of roots (about 5 mm from the root apex) showed that the diameter of roots in this region were increased, accompanied by cortical cell radical enlargement and distortion after stressed with 200 mM NaCl for 48 h and 96 h as compared with the control group (Figure 2A-D). In the control group, epidermal and cortical cells were isodiametric and uniformly placed (Figure 2A, C), whereas in the stressed plants the shape and distribution of epidermal and cortical cells were irregular (Figure 2B, D). The size of the cortical cells was slightly increased after treatment with 200 mM NaCl for 48 h but greatly increased for 96 h and accompanied by cortical cell radical enlargement. Furthermore, the number of cortical cell layers was not changed in the treated seedlings for 48 h and 96 h, but the number of the stele tissue cell layers was increased (Figure 2B, D). The increase of the cortex in the width must have predominantly been due to the cortical cell radical enlargement, which concomitantly caused the root swelling, which might be adaptive responses of plants to high-salinity stress.

**Figure 3:**
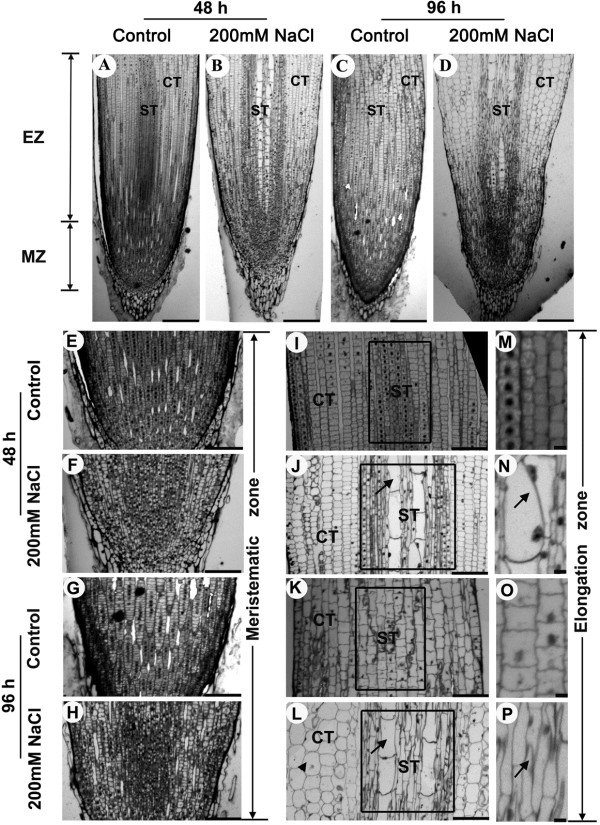
**Light-microscopy images of MB-stained longitudinal sections.** Longitudinal sections of six-day-old maize root tips grown under control conditions **(A, C)** or in the presence of 200 mM NaCl **(B, D)** in nutrient solution for 48 h **(A, B)** and 96 h **(C, D)**. **E-P** Magnification images of MB-stained longitudinal sections through the root tips of a non-stressed plant **(E, I, M, G, K, O)** or a plant stressed with 200 mM NaCl **(F, J, N, H, L, P)** for 48 h **(E, I, M, F, J, N)** and 96 h **(G, K, O, H, L, P)**. **E-H** Longitudinal sections of the meristematic zone from the root tips. **I-P** Longitudinal sections of the elongation zone from the root tips. The black rectangular frames in **I**-**L** indicate stele tissue observed. Arrows in **J** and **L** indicate transverse and radial enlargement cells observed in stele tissues. Arrowhead in **L** indicates radical enlargement cell observed in cortex. Arrows in **N** and **P** indicate irregular cell-division planes in stele tissue observed. Photographs were taken under bright-field illumination. MZ - meristematic zone; EZ - elongation zone; CT - cortex; ST - stele tissue. Bar = 0.25 mm **(A-B)** or 0.1 mm **(E-P)**.

We also used root longitudinal sections to analyze effects of NaCl on roots. The longitudinal sections of the roots (0–3 mm from the root apex) were observed after 48 h and 96 h of treatment with 200 mM NaCl. Root growth is a consequence of cell division in the meristematic zone and cell elongation in the elongation zone. According to root morphology and Feulgen staining, above the root cap is the meristematic zone (MZ) and the elongation zone (EZ) is located between the MZ and the root hair zone. The diameter of the longitudinal section of the root was increased especially in the elongation zone after 200 mM NaCl treatment (Figure 3A-D). After 48 h of treatment with 200 mM NaCl, the width of cortex was almost not changed, but the width of the stele tissue was increased (Figure 3A, B). After 96 h of treatment, the width of cortex and stele tissue was dramatically increased (Figure 3C, D). The root cells were vertical alignment with almost uniform size for the cortex and stele tissue in the control group (Figure 3A, C), but messed alignment with totally different size in cortex and stele tissue in the stressed plants (Figure 3B, D). The meristematic zone cells are applanate with a bigger size and aligned in control plants (Figure 3E, G). In contrast, the meristematic zone cells were arranged disorderly with a smaller size but with increased cell numbers after subjected to high-salinity stress (Figure 3F, H). The cell proliferating activity was reduced, which was verified by Feulgen staining (Figure 1B). Root elongation growth is dependent on massive expansion of cells continuously produced by meristematic tissues at the root tip; inhibition of the root growth by salinity is associated with an inhibition of this cell expansion [2,29]. Thus, the reduction of cell division activity and the inhibition of meristematic zone cells to expand to elongation zone cells may cause the inhibition of root growth. After 48 h of treatment, the number of cortical cell layers and the size of the cortical cell were almost not changed, but the number of stele tissue cell layers were increased, which was highlighted by a black rectangular frame, and accompanied by transverse and radial enlargement of the cells emerging in the stele tissue pointed out by an arrow (Figure 3I, J). After 96 h of treatment, the number of cortical cell layers was almost not changed, but the cortical cell radial enlargement was observed, and the total number of cortical cells per column of cells in the elongation zone was decreased compared to the control group (Figure 3K, L). The number of the stele tissue cell layers was increased after treatment which was pointed out by a black rectangular frame, accompanied by transverse and radial enlargement of the cells emerging in stele tissue pointed out by an arrow (Figure 3K, L). This result was consistent with that at 48 h (Figure 3I, J). Furthermore, control plants showed well-organized stele tissues with almost horizontal cell division planes in the elongation zone (Figure 3M, O), but the stressed plants exhibited irregular cell-division planes (Figure 3N, P) which might increase the number of stele tissue cell layers.

### High salinity activates the expression of HATs and increases global histone acetylation levels in the genome

**Figure 4:**
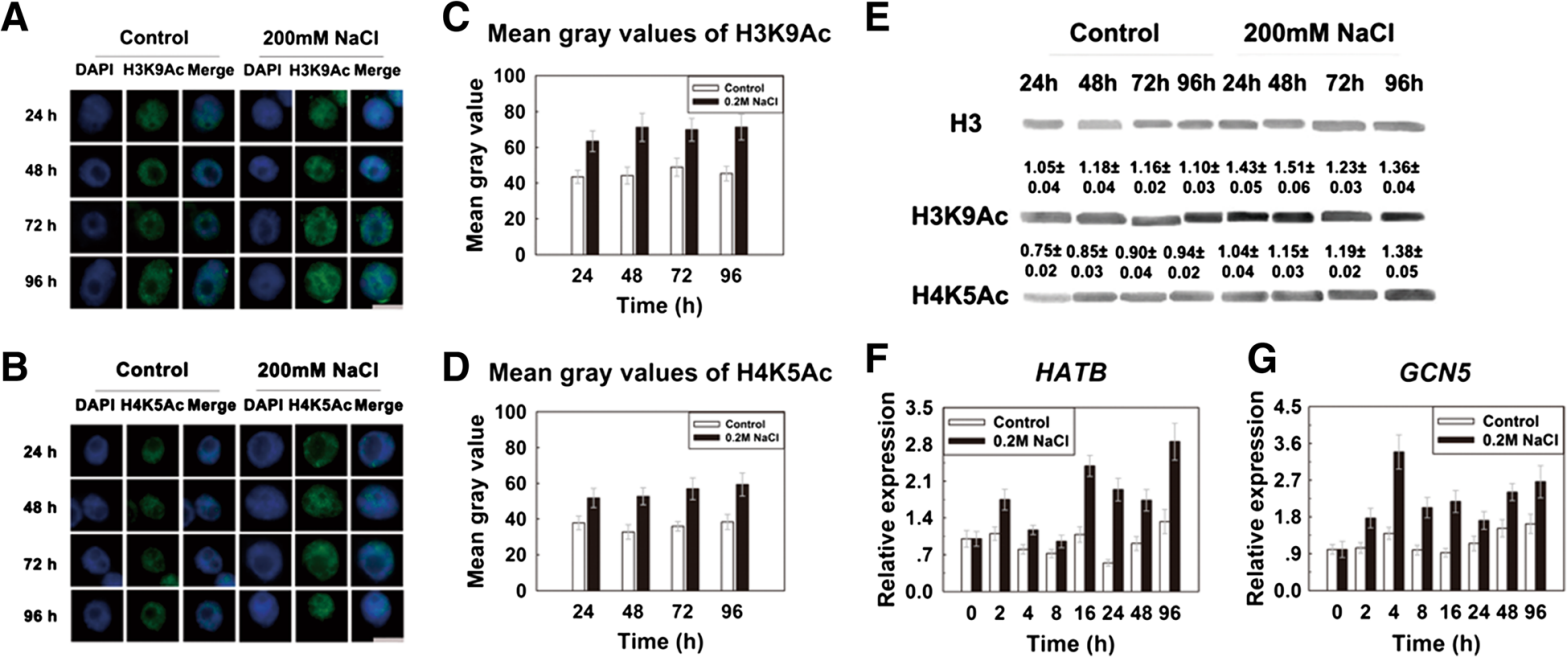
**High-salinity increased histone acetylation levels. (A, B)** The histones H3K9 and H4K5 acetylation levels were increased after 200 mM NaCl treatment. Nuclei from six-day-old seedlings untreated (Control) or treated with 200 mM NaCl for 24, 48, 72 and 96 h were subjected to immunofluorescence using antibodies against H3K9Ac **(A)** and H4K5Ac **(B)**. The ‘DAPI’ panel displays DAPI-stained DNA images pseudo-colored with blue, the ‘H3K9Ac’ and ‘H4K5Ac’ panels show immunostained images pseudo-colored with green, and the ‘Merge’ panel indicats a combination of blue and green signals. Bar = 10 μm. **(C, D)** Histogram displaying the mean gray values of the immunostaining signals for H3K9Ac **(C)** and H4K5Ac **(D)**. The mean gray values for H3K9Ac and H4K5Ac were increased by approximately 40% to 60% after treatment with 200 mM NaCl. More than 300 nuclei were analyzed and error bars represent the standard error of the mean gray value. **(E)** High-salinity affects H3K9Ac and H4K5Ac protein levels. The antibodies specific for H3K9Ac and H4K5Ac were employed to analyze histones extracts from seedlings untreated (Control) and treated with 200 mM NaCl for 24, 48, 72 and 96 h. Histone H3 was applied as an equal loading control. The average values of abundance index of H3K9Ac and H4K5Ac were indicated in each lane (Mean ± SD). After treatment with 200 mM NaCl, an increase in global H3K9Ac and H4K5Ac were observed at the indicated times. **(F, G)** Expression patterns of *ZmHATB***(F)** and *ZmGCN5***(G)** genes at different time points were determined by RT-PCR in maize seedlings under salt stress conditions. The y-axis indicates relative expression value and the x-axis indicates the hours after salt stress. The relative quantity was calculated using the *beta actin* gene as an internal reference. The relative expression value of 0 h was assigned as 1. Each experiment was repeated three times and relative expression values are shown as the average values ± SD (the standard deviation). White bars, no treated seedlings; Black bars, seedlings treated with 200 mM NaCl.

Recent studies have demonstrated that histone acetylation of chromatin is involved in plant responses to drought and cold stress [30,31]. To investigate total dynamic changes in histone acetylation under salt stress in maize roots, we carried out in situ chromatin immunostaining of interphase nuclei prepared at various time points using commercially available antibodies to H3K9Ac and H4K5Ac. As shown in Figure 4A and Figure 4B, in the control groups the signals in nuclei for the histones H3K9 (Figure 4A) and H4K5 (Figure 4B) acetylation were not significantly altered under normal growth conditions, but in contrast, acetylation signal intensity was increased after treatment with 200 mM NaCl compared to the control groups, indicating that the acetylation levels of H3K9 and H4K5 were increased under salt stress. Quantification of the signal intensity of mean gray values showed that the H3K9Ac and H4K5Ac levels were increased by approximately 40% to 60% after 200 mM NaCl treatment (Figure 4C, D). We further performed western blot detection of H3K9Ac and H4K5Ac in the untreated and treated seedlings with 200 mM NaCl. The results showed that the H3K9 (1.05-1.18) and H4K5 (0.75-0.94) acetylation levels under normal growth conditions were not significantly altered at the indicated times, but salt stress induced an increase in global acetylation of H3K9 (1.23-1.51) and H4K5 (1.04-1.38) as the duration of exposure was increased (Figure 4E). It is known that histone acetylation is catalyzed by HATs [25]. Thus we analyzed HAT expression pattern in maize roots treated with and without 200 mM NaCl using RT-PCR. Two HAT genes (*HATB* and *GCN5*) were selected from two types of HATs (HAT-A and HAT-B). Using quantitative real-time PCR after reverse transcription of RNA, we found that mRNA levels of the *ZmHATB* and *ZmGCN5* genes were increased from 2 to 96 h in response to salt treatment (Figure 4F, G). In untreated seedlings, the mRNA levels of the *ZmHATB* and *ZmGCN5* genes were almost not increased during growth process. By comparison, the transcript level of *ZmHATB* (Figure 4F) reached the maximal value at 96 h which was 2.8, while the transcript level of *ZmGCN5* (Figure 4G) reached the maximal value at 4 h which was 3.3.

### High salinity selectively affects the expression of the cell-wall related genes

**Figure 5:**
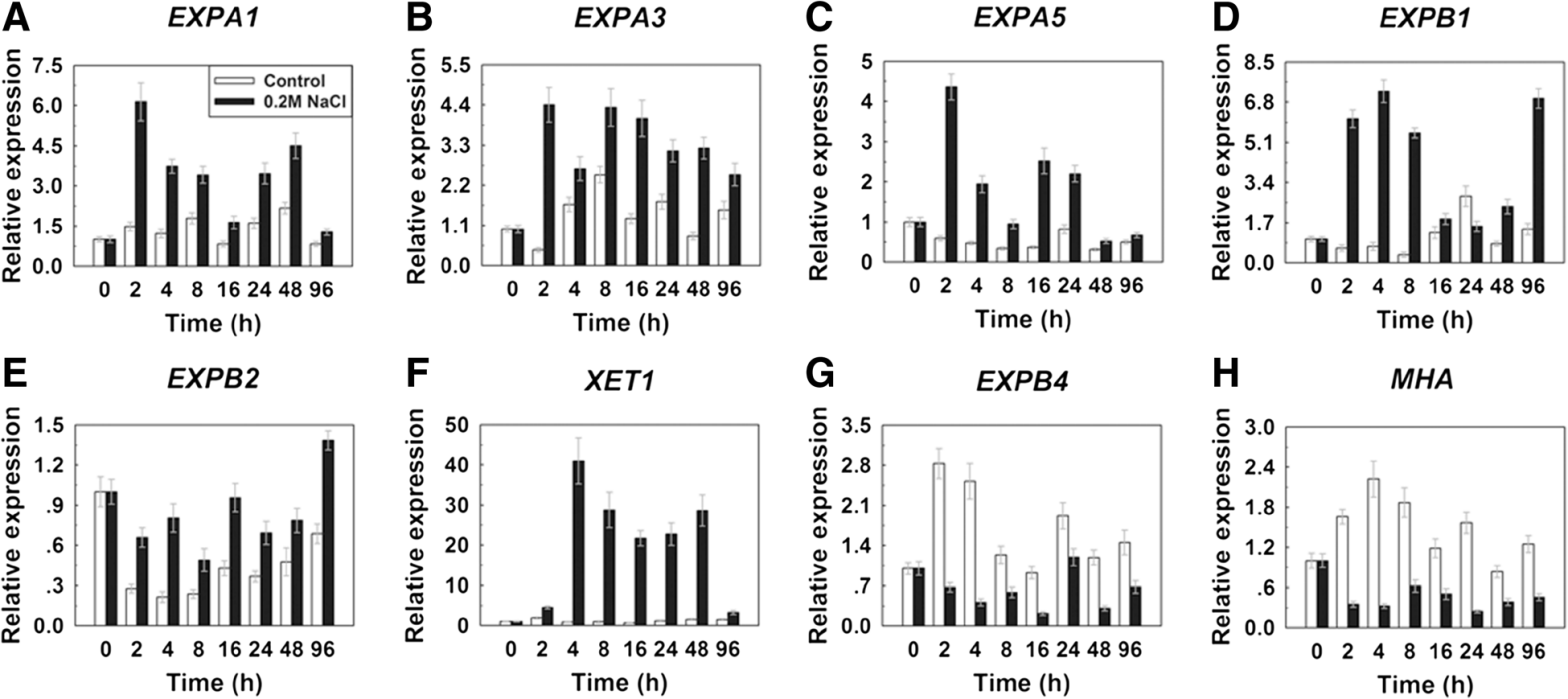
**The differential expression of cell wall related genes.** Quantitative real-time PCR (RT-PCR) analysis of α-expansins **(A-C)**, β-expansins **(D, E, G)**, *ZmXET1***(F)** and *ZmMHA***(H)** genes at different time points after treatment of six-day-old maize seedlings with 200 mM NaCl. Maize root samples were harvested after 0, 2, 4, 8, 16, 24, 48 and 96 h for RNA isolation. The y-axis shows relative expression values and the x-axis indicates the hours after treatment with NaCl. Expression values were normalized to those of the *beta actin* gene. The relative expression value of 0 h was assigned as 1. Each experiment was repeated three times and relative expression values are shown as the average values ± SD (the standard deviation). White bars, no treated seedlings; Black bars, seedlings treated with 200 mM NaCl.

Growth is a process of an increase in cell numbers and cell volumes. The cell enlargement is accomplished by simultaneous vacuolar enlargement and irreversible cell wall expansion [14]. Expansins are proteins involved in cell wall loosening [32]. XET has been proposed as a potential protein for cell wall extension [20]. The plasma-membrane proton pump (PM-H^+^-ATPase) can pump protons from the cytosol into the apoplast, resulting in cell wall loosening and cell expansion [19]. The above anatomy experiment showed that roots were swollen due to cell radial enlargement in the elongation zone after high salinity treatment, so we wanted to know whether the expression of these cell wall related genes was affected. To further analyze the temporal expression patterns of these genes, time course analysis by RT-PCR was performed. Six-day-old maize seedlings were exposed to 200 mM NaCl, and maize root samples were harvested after 0, 2, 4, 8, 16, 24, 48 and 96 h for RNA isolation. The mRNA levels of the tested genes were normalized with respect to the level of the *beta actin* gene, whose transcription level was stable in maize under salt stress [33,34]. Previous work identified four expansin genes highly expressed in the maize roots, namely the two α-expansins, *ExpA1* and *ExpA5*, and two β-expansins, *ExpB2* and *ExpB4*[35]. The expression pattern of *ZmExpA3* is not consistent with that of *ZmExpA1*, and *ZmExpA3* has a role in wall loosening for shoot cell elongation under salt stress [12]. *ExpB1* is a gene particularly expressed in pollen, and as one of the group-1 allergens, has a wall-loosening role, aiding penetration of the pollen tube through the stigma and style by softening the maternal cell walls [36]. So we analyzed the expression of three α-expansin genes, *ZmExpA1*, *ZmExpA3* and *ZmExpA5*, three β-expansin genes, *ZmExpB1*, *ZmExpB2* and *ZmExpB4*, and the *ZmXET1* and *ZmMHA* genes in maize roots. Our data showed that the transcript levels of *ZmEXPA1*, *ZmEXPA3*, *ZmEXPA5*, *ZmEXPB1*, *ZmEXPB2* and *ZmXET1* were remarkably increased from 2 to 96 h after exposure to high-salinity treatment (Figure 5A-F). The *ZmEXPA1*, *ZmEXPA3*, *ZmEXPA5*, *ZmEXPB1* and *ZmEXPB2* mRNA levels began to substantially accumulate after 2 h of salt stress (Figure 5A-E), and the *ZmEXPA1*, *ZmEXPA3* and *ZmEXPB2* mRNA levels (Figure 5A, B, E) were substantially and steadily increased from 2 h to 96 h after the treatment, while *ZmEXPA5* (Figure 5C) was substantially increased from 2 h to 24 h after the treatment but slightly increased from 48 h to 96 h. The *ZmEXPB1* (Figure 5D) were substantially increased at 2, 4, 8, 96 h and slightly increased at 16, 48 h but decreased at 24 h. The *ZmXET1* transcript level (Figure 5F) was substantially increased from 4 h to 48 h after salt stress but slightly increased at 2 h and 96 h, which was 40-fold higher than untreated seedlings at 4 h. In contrast, *ZmEXPB4* and *ZmMHA* mRNA levels were decreased from 2 to 96 h after the treatment (Figure 5G, H).

### The local H3K9Ac levels of *ZmEXPB2* and *ZmXET1* were increased under high-salinity stress

**Figure 6:**
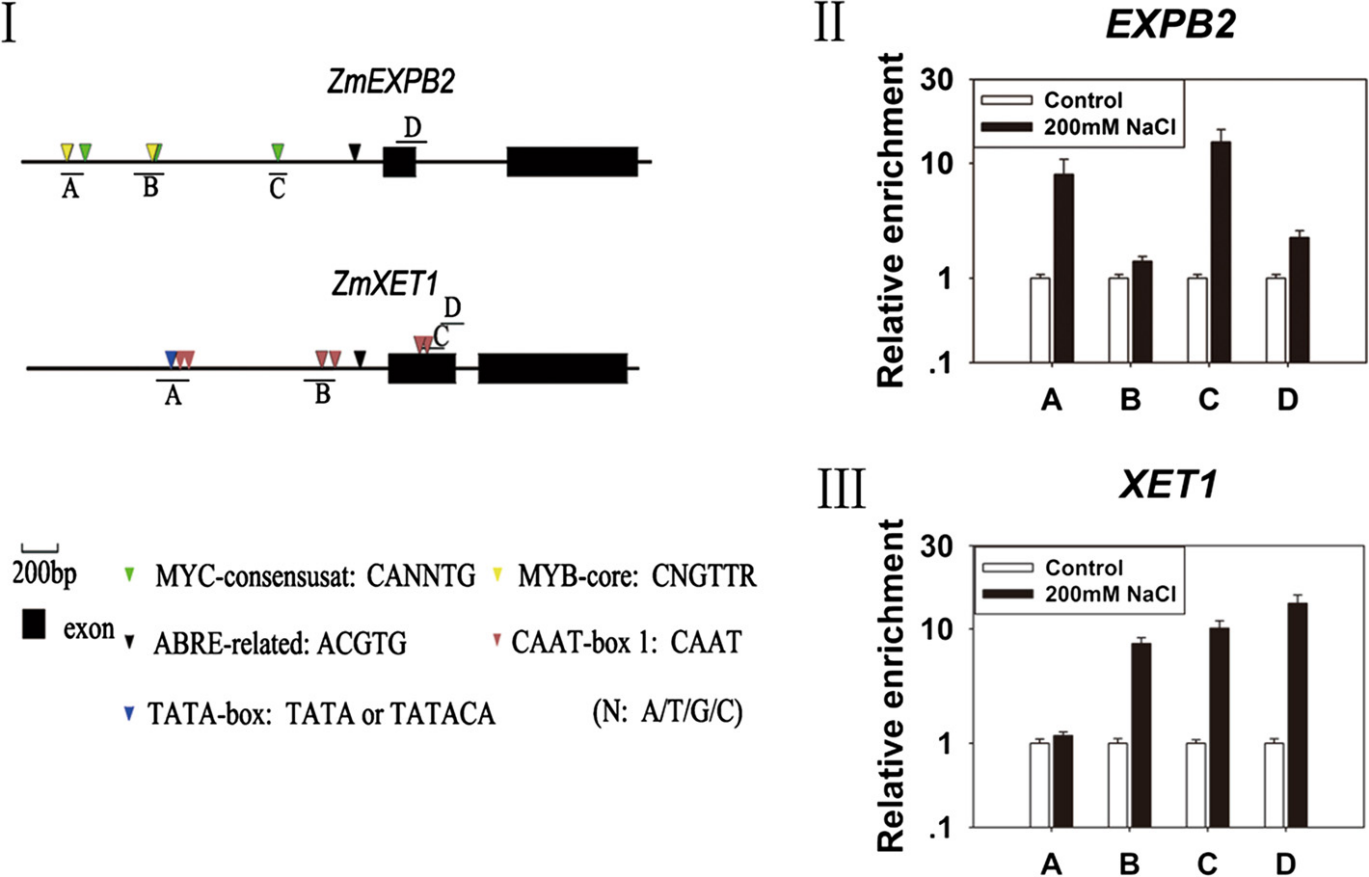
**The elevated H3K9 acetylation levels on *ZmEXPB2 *and *ZmXET1 *gene regions. (I)** Schematic diagram of *ZmEXPB2* and *ZmXET1* gene regions and their amplification positions for ChIP analysis. Exons are marked by black boxes. In this study, we regarded the 5’ end sequence of the first exon as the translation start site (+1). The following cis-acting element sequences in the PLACE 26.0 database ( http://www.dna.affrc.go.jp/PLACE/) are indicated as: green triangle, MYC-consensusat: (CANNTG); yellow triangle, MYB-core (CNGTTR); black triangle, ABRE-related (ACGTG);purple red triangle, CAAT-box1 (CAAT); blue triangle, TATA-box: (TATA or TATACA). N represents A, T, C or G. Bars underneath the sequence marked as **A, B, C, D** respectively represent the positions of the PCR-amplified regions for ChIP assay. The following regions were PCR amplified using the region-specific primer sets (Table 2). **(II, III)** Alterations of H3K9Ac enrichment on *ZmEXPB2***(II)** and *ZmXET1***(III)** genes after treatment of six-day-old seedlings with 200 mM NaCl for 48 h. The relative H3K9Ac levels were determined by ChIP assays using an antibody to H3K9Ac, and normalized to an internal control gene *beta actin*. Letters on the x-axis on each graph represent the positions of the PCR-amplified fragments (shown in **I**). The y-axis values represent the relative enrichment of histone modification. The relative enrichment value for each pair of primers in the control group was assigned as 1. Each experiment was repeated three times and relative acetylation levels are shown as the average values ± SD (the standard deviation). White bars, no treated seedlings; Black bars, seedlings treated with 200 mM NaCl.

The transcript levels of *ZmEXPB2* and *ZmXET1* were increased after treatment with 200 mM NaCl for 48 h. It is generally accepted that histone acetylation is generally associated with gene transcription [37]. To determine whether the change of the transcript levels of *ZmEXPB2* and *ZmXET1* at 48 h under salt stress was due to the alteration of histone modifications, we performed ChIP experiments using an antibody against at histone H3 acetylated at K9 (H3K9Ac) on the maize roots untreated and treated with 200 mM NaCl for 48 h. Four different regions of the *ZmEXPB2* and *ZmXET1* genes were selected to conduct ChIP experiments (Figure 6). For *ZmEXPB2* gene, the acetylation levels were substantially increased on promoter regions A and C, and slightly increased on the promoter region B and the coding region D (Figure 6). For *ZmXET1* gene, the acetylation levels were substantially increased on the promoter region B and the coding regions C and D, and slightly increased on the promoter region A (Figure 6).

## Discussion

### High salinity inhibited root growth and resulted in cell enlargement and root swelling

A high concentration of NaCl reduced root growth in many crop plants [38]. In this study, 200 mM NaCl treatment caused maize growth inhibition, and the primary root length was significantly reduced. This was consistent with previous observations in maize and cotton seedling roots [2,39]. The swelling elongation zone became wider and longer and the meristematic zone was reduced in length with the increasing of the treatment time with 200 mM NaCl (Figure 1B). Root swelling was also observed in maize roots exposed to salt stress [40] and aluminium stress [41,42]. The formation of tuberized roots also has been reported in *A.thaliana* to be a consequence of drought stress [43] and salt stress [44,45]. It has been reported the length of the meristematic zone of the primary root tips was reduced by 56% after 1 week of 1% NaCl treatment in *A.thaliana*. Our cytological analysis showed that the cortical cell radical enlargement after 200 mM NaCl treatment resulted in an increase in the root diameter. Similarly, a significant decline in the ratio of the cross-sectional area of the stele to area of the root was observed with increasing NaCl concentration in cotton roots [39]. It has been reported that a radical swelling of all cell layers in root tips of *Arabidopsis thaliana* after 2 weeks of 1% NaCl stress [44]. The length and volume of the cortical cells were increased, but the cell density in the cortex was significantly decreased, indicating that the cell production was decreased during salt stress. It has been reported that in cotton roots salinity diminished the rate of cell production [39]. Burssens et al. [44] reported that the inhibition of *A.thaliana* root growth with salt stress is at least partially due to a decrease of cell production. The stele tissue cells transport water and soluble mineral nutrients from the roots to shoots. The transverse and radical enlargement of stele tissue cells was first emerged on the longitudinal section and the number of stele tissue cell layers was increased both in the transverse section and longitudinal section of the roots. These may help cells uptake more water and create more barrier to reduce Na^+^ concentration, which may be an adaptive mechanism to defense ionic toxicity. A decrease in the meristematic zone length of the primary root and the elongation zone cell numbers may be the reasons why the root growth was inhibited.

### High salinity induced cell enlargement and root swelling in the elongation zone are accompanied by up-regulation of some cell wall related genes

The expression levels of expansin genes were increased in response to submergence in deepwater rice internode [46]. The *GmEXP1* expression level was very high in soybean roots where rapid root elongation took place and ectopic expression of *GmEXP1* accelerated the growth of transgenic tobacco roots, which showed insensitivity to stress [18]. Excised stem segments treated with auxin rapidly increased cell elongation, and the mRNAs of *EXPA1*, *EXPA3*, *EXPA4* and *EXPA5* were increased within 1 h [47]. *ExpB2* plays a role in the elongation of maize roots, and may be also involved in plant responses to environmental stimuli [48]. It has been reported that the expression of *EXP1*, *EXP5*, *EXP6* and *EXP8* genes was up-regulated in maize primary roots after grown at low ψ_w_, which likely contributed to enhanced cell wall extensibility and thus helped root cells maintain elongation at reduced turgor pressure [35]. The expression of XET and expansins was about 100-fold higher in cotton fiber cells, corresponding to their proposed role in cell enlargement [49]. The transcription levels of expansins and XET were increased after salt stress [50,51]. The XET activity was enhanced in the apical region of maize roots from plants grown under low water potentials, and was suggested to be necessary for maintaining elongation [52]. Our RT-PCR experiment showed that the transcript levels of *ZmEXPA1*, *ZmEXPA3*, *ZmEXPA5*, *ZmEXPB1*, *ZmEXPB2* and *ZmXET1* were increased from 2 to 96 h after exposure to high-salinity treatment. We presumed that the up-regulation of these five expansin genes and *ZmXET1* was an adaptive mechanism to regulate the transverse and radical enlargement of the elongation zone cells, which may mitigate the decrease in root growth and the damage under high-salinity stress. It has been reported that the average cell length of mesocotyls was increased by up to 58% in the transgenic lines that overexpressed *OsEXP4*[8]. The *ZmEXPB4* gene was down-regulated after treatment with 200 mM NaCl. Hormone treatment induced expression of *Exp1* but repressed that of *ExpB2* in maize roots [30]. Therefore the differential expression patterns of expansins suggest that each expansin member may play a specific role in root growth and development, and in response to external stimuli. The acid growth theory thought that an auxin-induced acidic environment was needed for elongation growth [53]. PM-H^+^-ATPase can pump protons into the apoplast from the cytosol to create a acidic environment and thus the down-regulation of *ZmMHA* may reduce root elongation growth.

### Histone acetylation may be involved in high salinity-induced gene expression regulation

Recent studies have revealed that gene expression is regulated by dynamic histone modification, which could be an important mechanism for plants to adapt to abiotic stress [31,54]. In tobacco BY2 and *Arabidopsis* T87 cells, high-salinity and cold stress triggered rapid up-regulation of histone H3 Ser-10 phosphorylation and histone H4 acetylation that was correlated with activation of stress-responsive genes [27]. The mutations of GCN5 and ADA2 that encode the components of histone acetyltransferase complexes affected the expression of the cold stress-responsive genes in *Arabidopsis*[55]. The enrichment of H3K9 acetylation and H3K4 trimethylation was related with the up-regulation of *RD29A*, *RD29B*, *RD20*, and *RAP2.4* genes in response to drought treatment [30]. Similarly, salt stress enriched H3K9K14 acetylation and H3K4 trimethylation on the promoter and coding regions of *DREB2A*, *RD29A*, and *RD29B*[26]. Our results showed that the total acetylation levels of H3K9 and H4K5 in the genome were increased after treatment with 200 mM NaCl and this increase could be associated with the enhanced expression of *ZmHATB* and *ZmGCN5*. Therefore overall histone acetylation level change is likely to be an adaptive response to salt stress at the epigenetic levels. It has been reported that the overall acetylation level alteration may be related with basal transcription and help rapid restoration of the acetylation level when the recruited HAT is removed [56]. Our results diaplayed salt stress caused the up-regulation of *ZmEXPB2* and *ZmXET1* genes, which was accompanied with the elevated H3K9 acetylation levels on promoter regions and coding regions of these two genes. These data support the conclusion that epigenetic regulation plays a vital role in rapid regulation of gene expression in plant adaptive response to environmental stimuli [57,58].

## Conclusions

This study showed that the stele tissue and cortex cells were enlarged after treatment with 200 mM NaCl, which was associated with an up-regulation of cell wall related genes *ZmEXPA1*, *ZmEXPA3*, *ZmEXPA5*, *ZmEXPB1*, *ZmEXPB2* and *ZmXET1*. The expression of histone acetyltransferase genes *ZmHATB* and *ZmGCN5* was increased accompanied by an increase in the global acetylation levels of histones H3K9 and H4K5, suggesting that epigenetic regulation was involved in salt stress response. ChIP experiment further indicated that the up-regulation of *ZmEXPB2* and *ZmXET1* genes was associated with the elevated H3K9 acetylation levels on promoter regions and coding regions of these two genes. These data imply that an epigenetic control of the expression of the cell wall related genes in response to salt stress results in cell enlargement and root swelling which is an adaptive response.

## Methods

### Plant materials and treatments

Maize seeds (*Zea mays* L. hybrid line Huayu 5) were germinated in the dark at 25°C on cotton gauzes soaked in water on the glass dish, and then the seedlings of uniform size were transferred to hydroponic cultures in buckets containing 1/2 Hoagland’s nutrient solution in a controlled environment chamber under relative humidity of 70%, photoperiod of 14 h irradiance of 120 μmol m^**-**2^ s^**-**1^ with temperatures of 25°C and in the dark 10 h of 20°C respectively. The solutions were fully renewed every 2 days. After 6 days, when the seedlings with two leaves, 200 mM NaCl was added to nutrient solution to initiate the saline treatment. Six-day-old maize seedlings grown in 1/2 Hoagland’s nutrient solution without NaCl were considered as a control group.

### Growth measurement

Six-day-old maize seedlings were transferred to nutrient solution supplemented with 0, 25, 50, 100, 150, 200 and 250 mM NaCl respectively for 7 days, then the image was obtained by Nikon J1 (Nikon Corporation, Japan). Six-day-old maize seedlings (n = 20) were transferred to nutrient solution supplemented with or without 200 mM NaCl treatment for 0, 2, 4, 8, 16, 24, 48 and 96 h, then maize seedlings were photographed by Nikon J1 (Nikon Corporation, Japan) and the primary root length and plant height were measured by Image J.

### Root swelling and Feulgen staining

Feulgen staining of the primary roots was performed on 20 maize seedlings after 24, 48, 72 and 96 h treatment with nutrient solutions containing 0 (control) or 200 mM NaCl. Primary roots were fixed over night in a solution of ethanol and glacial acetic acid in a 3:1 ratio. Subsequently, roots were washed several times with 70% ethanol, followed by a gradual rehydration in increasing ethanol concentrations, 5 min per step with three changes of water at the end [59]. Hydrolysis was performed in 1 N HCl for 15 min at 60°C, and stopped by replacing HCl with water. Root staining was achieved for 1 h in the dark at room temperature with Schiff’s Solution (Sigma, Taufkirchen, Germany) [60]. After 1 h the roots were washed three times by deionized water and examined by Stereo Microscope (China) with 10X objective and 0.8X ocular. Images were captured by IScapture software (ISC, China) with a CCD monochrome camera (TCC-5.0, China).

### Light microscopy

For light microscopy studies, after a short rinse (10 s) with distilled water, the tips (0–10 mm) from primary roots were excised from control and 200 mM NaCl treated seedlings after 48 h and 96 h of exposure to salt treatment. The samples were immediately fixed with 3% glutaraldehyde and post fixed with 1% osmium tetroxide, dehydrated in ethanol series followed by embedded in Spurr’s resin. The transverse sections at approximately 5 mm from the apex and the longitudinal sections between 0 and 3 mm from apex were cut by ultramicrotome. Semi-thin transverse and longitudinal sections were stained with methylene blue (MB). Methylene blue stained specimens were examined with an Olympus BX-60 fluorescence microscope (Olympus, Tokyo, Japan) with bright-field illumination at 4X and 10X. Images were captured with a CCD monochrome camera Sensys 1401E and processed with ADOBE PHOTOSHOP 9.0 software (Adobe Systems, San Jose, CA).

### Immunostaining

The nuclei of maize roots were prepared in the slides according to the reported method [61]. Immunostaining of the nuclei on the slides was carried out as described by Zhang et al. [62]. The primary antibodies were H3K9Ac (catalog number: 07–352, Millipore, Billerica, MA, USA) and H4K5Ac (catalog number: 06–759, Millipore, Billerica, MA, USA) and the secondary antibody was fluorescein conjugated goat anti-rabbit IgG (catalog number: 12–507, Millipore, Billerica, MA USA). In control experiments, slides were incubated with the secondary antibody only. All slides were counterstained with 4,6-diamidino-2-phenylindole (DAPI, Sigma, USA), mounted with Vectashield (Vector labs, USA). Images were captured with a CCD monochrome camera Sensys 1401E under an Olympus BX-60 fluorescence microscope with filter blocks for DAPI and fluorescein, then pseudo-colored and merged using the software MetaMorph 7.7.2 (Universal Imaging Corp., USA). Microscope settings and camera detector exposure times were kept constant for the control and treated groups and more than 300 nuclei were analyzed. Images were processed using ADOBE PHOTOSHOP 9.0 software (Adobe Systems, San Jose, CA). The mean gray value of the immunostaining signals for H3K9Ac and H4K5Ac in the control and NaCl-treated samples was measured with Image J and MetaMorph. For both the control and treated groups, three independent immunostaining experiments were performed with each antibody. Mean gray value of the signal intensity and standard error of the mean value were calculated with SPSS10.0 for Windows package (SPSS Inc., 1999).

### Western blot assay

Proteins were extracted from maize seedling roots by grinding in the liquid nitrogen and resuspended in the extraction buffer [100 mM Tris–HCl pH 7.4, 50 mM NaCl, 5 mM ethylenediaminetetraacetic acid (EDTA) and 1 mM phenylmethanesulfonyl fluoride (PMSF)]. Western blot detection was carried out as described by Yang et al. [63]. Proteins were fractionated by SDS-PAGE and transferred to Immobilon-P membranes which were respectively incubated with the primary antibodies H3 (catalog number: 06–755, Upstate, Lake Placid, NY, USA), H3K9Ac and H4K5Ac overnight at 4°C. Detection was performed using alkaline phosphatase (AP) conjugated anti-rabbit IgG antibody and chemiluminescence visualization. Histone H3 was applied as an equal loading control. Densitometric measurements were taken after immunodetection using Image J. Abundance index was calculated as follows: H3K9Ac or H4K5Ac band intensity/H3 band intensity. Western blots were repeated three times for each sample from three independent experiments. Mean abundance index and standard error of the mean were calculated with SPSS10.0 for Windows package (SPSS Inc., 1999).

### Quantitative real-time PCR

Total RNA was isolated with Trizol reagent (Invitrogen, USA). The purified RNA was reverse-transcripted to cDNA by using RevertAid First Strand cDNA Synthesis Kit (Fermentas, Burlington, ON, Canada). The reverse transcription product was diluted by ten times to perform real-time PCR amplification reaction in triplicate for technical repeats. Quantitative real-time polymerase chain reaction (RT-PCR) was carried out using SYBR® Green Real-time PCR Master Mix (TOYOBO, Tokyo, Japan) in an ABI StepOne Plus real-time PCR system (Applied Biosystems, Carlsbad, USA) with the following cycling conditions: 94°C for 1 min, followed by 40 amplification cycles at 94°C for 15 s, 56°C for 30 s and 72°C for 30 s. Fluorescence data were acquired at the 72°C step and during the melting-curve program. The threshold cycle numbers (Ct) for each PCR products were determined, and the relative expression levels for all genes were obtained using the 2^-△△Ct^ calculation. To measure the transcript levels of four composite *Zea mays* plasma-membrane H^+^-ATPases (*ZmMHA1*, *ZmMHA2*, *ZmMHA3*, and *ZmMHA4*), we used a degenerated primer pair for the *ZmMHA* gene designed by Geilfus et al. [12]. *Zea mays* xyloglucan endotransglucosylase homolog 1 (*ZmXET1*) was used as a representative to study xyloglucan endotransglucosylase. Quantitative RT-PCR primers were designed by Primer-BLAST (NCBI) to amplify about 200 bp fragments. Preliminary experiments were done to ensure the amplification of a single PCR product for the analyzed gene and standard curves were generated for each primer set to determine their efficiency. The sequences and the PCR efficiencies of the primers used for quantitative RT-PCR are listed in Table 1. In the preliminary experiment, we also tested the expression of three common reference genes such as *Actin* (GRMZM2G126010_T01), *18S rRNA* (GRMZM2G114613_T02) and *UBQ* (GRMZM2G118637_T02), in the control and 200 mM NaCl treated seedlings, and we observed that *actin* transcription was the most stable as the CT value of *actin* did not vary much at the same content of DNA template between the control and treated groups (Ct values: 25.3236 ± 0.2394). The *beta actin* gene was used as a reference gene, also because some publications reported that its expression in maize roots is little affected by salt stress [33,34]. RT-PCR was repeated three times for each sample from three independent experiments.

**Table 1:** **RT**-**PCR primer pairs for maize** (***Zea mays *** **L**.)

**Primer**	**Sequence (5'-3')**	**Efficiency**
*ZmHATB*	CAGCTGACCTGATGGAGACT (F)	98%
	TTGGCATCTGCAACAGACGC (R)	
*ZmGCN5*	GGACGGCTGAAGTTTCTCTG (F)	97%
	GCTTGCATAAGGGCGATAAG (R)	
*ZmXET1*	CTACCAGGACGTGGACATCA (F)	99%
	ACCCTGCGACGAAAGATAGA (R)	
*ZmMHA*	AGCCAGGCYCTKATCTTCGT (F)	101%
	SACGATGYTGTASAGCCAGA (R)	
*ZmEXPA1*	CGAGTGTGACTGTGAGCAAGAGA (F)	102%
	GCCTTGCAGGTTTTTGTTTTG (R)	
*ZmEXPA3*	CATCATCAGTCTCTCGCGCT (F)	96%
	TACCCCGTCGTCGAGTACAGGTT (R)	
*ZmEXPA5*	GCCAGTTCTGAGGATGAACAGC (F)	98%
	TGGTCCGATCCAGTCCGTAA (R)	
*ZmEXPB1*	CTACACTTCCAACGTCCAATTCTACT(F)	99%
	TTCGATCATGAACCCGAACA (R)	
*ZmEXPB2*	CACCACCCACCACTACTACCA (F)	102%
	AACGACTCAAAGGACCATGACAA (R)	
*ZmEXPB4*	ACCATCGTAATCACCGACCA (F)	103%
	CTTCAACCTCCTCTTTCATTCTCT (R)	
*ZmActin*	AAACGGCTACCACATCCAAG (F)	101%
	CCTCCAATGGATCCTCGTTA (R)	

### Chromatin immunoprecipitation (ChIP)

ChIP assays were performed using standard procedures [64]. Six-day-old maize seedlings were further grown in 1/2 Hoagland’s nutrient solution with or without 200 mM NaCl for 48 h and 20 g fresh samples were ground to powder in liquid N_2_, suspended in TBS buffer, then filtered, washed twice by different concentration of sucrose solution, and centrifuged. Equal amounts of chromatin extract was digested into 200–500 bp with micrococcal nuclease (1U/ul, Sigma) at 37°C for 10 min. Chromatin was precleared with protein A-sepharose (Upstate, Lake Placid, NY) at 4°C for 3 h and then incubated over night at 4°C with 10 μl anti-H3K9Ac (Millipore, MA, USA. #07-352) and 10 μl rabbit serum. After immunoprecipitation, the extracts were gradient eluted by different concentrations of NaCl solutions, once with a low salt buffer (50 mM Tris–HCl pH 7.5, 10 mM EDTA, 50 mM NaCl), then once with a middle salt buffer (50 mM Tris–HCl pH 7.5, 10 mM EDTA, 100 mM NaCl), and finally once with a high salt buffer (50 mM Tris–HCl pH 7.5, 10 mM EDTA, 150 mM NaCl), and centrifuged. Subsequently, the precipitations were eluted twice with an elution buffer (50 mM NaCl, 20 mM Tris–HCl pH 7.5, 5 mM EDTA, 1% SDS) at 65°C for 15 min, and centrifuged to collect the supernatant. Next, the DNA in the supernatant was extracted with a standard procedure (phenol/chloroform/isoamyl alcohol) (25:24:1) to perform quantitative real-time polymerase chain reaction with the SYBR® Green Real-time PCR Master Mix (TOYOBO, Tokyo, Japan). The *beta actin* gene as a control gene was used for normalization of ChIP-QPCR [30,65,66], which can be reliably used with high quality measurements [64]. The extract precipitated with rabbit serum was used as a negative control. And the negative control immunoprecipitations with rabbit serum on both control and treated groups did not result in significant PCR product. So we only analyzed the immunoprecipitations with H3K9Ac antibodies on the control and treated groups For each primer pair, we calculated the relative enrichment of the amplified product with the comparative CT method according to Saffery et al. [67], using the gene *actin* as the reference. We calculated CT difference for control DNA as △CT (control DNA) = CT (gene of interest) -CT (*actin*) and that for treated DNA as △CT (treated DNA) = CT (gene of interest) -CT (*actin*). Then we calculated the relative enrichment as 2^-△△Ct^, where △△CT = △CT (treated DNA) -△CT (control DNA). The relative enrichment value for each pair of primers in the control group was assigned as 1. The amplification conditions were: 94°C for 1 min, followed by 45 amplification cycles at 94°C for 5 s, 56°C for 15 s and 72°C for 20 s. Quantitative real-time PCR were performed with the primers specific for Zm*EXPB2* and Zm*XET1* gene at different regions. The sequences, amplification loci and the PCR efficiencies of the primers used in this experiment are listed in Table 2. ChIP experiments were repeated three times for each sample in three independent experiments.

**Table 2:** **ChIP**-**PCR primer pairs for maize** (***Zea mays *** **L**.)

**Primer**	**Sequence (5'-3')**	**Efficiency**	**Product length (bp)**	**Start**	**Stop**
*ZmEXPB2* SetA	CTTCGCCTAAACGTGTGCTC (F)	99%	125	-1802	-1676
	CGTGCCAACCTAGCTACAGA (R)				
*ZmEXPB2* SetB	CACCCACCCATCTCACAGAC (F)	101%	170	-1396	-1225
	GCGCGTATTAAAAGCTGGCA (R)				
*ZmEXPB2* SetC	TTAGAGTAGCACGCCAACCG (F)	98%	100	-640	-539
	GGAGATCGAGTGAGAGACGC (R)				
*ZmEXPB2* SetD	TACCCGGTGTCCAAGTACCA (F)	97%	105	70	174
	GATGTCGATGATGCCCGAGT (R)				
*ZmXET1* SetA	GCATGGCACACATTGCACTA (F)	98%	186	-1300	-1115
	CGTTCGTGGCGCATTATTCA (R)				
*ZmXET1* SetB	CGGGCATTCGTTCGTTTTGT (F)	102%	179	-475	-297
	CGGCTCTTCATGATCCCTCC (R)				
*ZmXET1* SetC	CGTCCTGGTAGAAGTTGCCG (F)	96%	132	182	313
	TGAAGCCTGAAGTGAACCGA (R)				
*ZmXET1* SetD	CGATCGGTTCACTTCAGGCT (F)	99%	132	291	422
	GCTATAAATACCGCGGCTCG (R)				
*ZmActin*	GATGATGCGCCAAGAGCTG (F)	101%	104	340	443
	GCCTCATCACCTACGTAGGCAT (R)				

### Accession numbers

The accession numbers for the genes described in this paper are *HATB* [GRMZM5G851405_T02], *GCN5* [GRMZM2G046021_T01], *XET1* [GRMZM2G026980_T02], *MHA* [GRMZM2G148374_T01, GRMZM2G019404_T02, GRMZM2G450055_T01, GRMZM2G006894_T01], *EXPA1* [GRMZM2G339122_T01], *EXPA3* [GRMZM2G074585_T02], *EXPA5* [GRMZM2G019398_T01], *EXPB1* [GRMZM2G146551_T02], *EXPB2* [GRMZM2G021621_T02], *EXPB4* [GRMZM2G154178_T01], and *Actin* [GRMZM2G126010_T01].

## Competing interests

The authors declare that they have no competing interests.

## Authors’ contributions

HL conceived the study, carried out the experiments, performed the statistical analysis and drafted the manuscript. QZ, PW, HH participated in root morphology analysis. LZ, FG carried out the molecular studies and performed the statistical analysis. SY, JT participated in the immunostaining and helped to draft the manuscript. LL conceived and designed the study and drafted the manuscript. All authors read and approved the final manuscript.
